# 

**Published:** 1911-12-16

**Authors:** 


*To the Editor of* The Hospital.

### Appointments.

Dr. John James Martin has been appointed assistant medical officer to the Portsmouth Parish Infirmary.

Mr. E. Wilmot Smerdon, M.B., Ch.B., has been appointed assistant house surgeon to the Derbyshire Royal Infirmary.

Mr. W. J. Carr, B.A. Cantab, M.R.C.S., L.R.C.P., has been appointed house physician at the Evelina Hospital for Sick Children.

Mr. A. J. O. Wigmore, M.R.C.S., L.R.C.P., M.B. Bristol, Ch.B., has been appointed assistant house surgeon at the East Sussex Hospital, Hastings.

### Florence Nightingale Memorial.

We have received the following contributions since those acknowledged last week. They are for a statue, unless otherwise indicated :—

Glasgow Western Infirmary (per Miss Gregory-Smith), £26 5s., instead of £25 acknowledged last week; viz. Cooperation of Trained Nurses, Glasgow (per Miss Rough), £9 13s. 6d. ; Matron and Nursing Staff, Western Infirmary, £9 1s. 6d. ; Sir George T. Beatson, K.C.B., £1 1s. ; Miss Florence Beatson, £1 1s. ; Mrs. J. J. Burnet, £1 ; James MacPherson, Esq., £1 ; Col. Roxburgh, £1 ; Matron and Nursing Staff, Knightswood Hospital, 14s. ; Nicol Paton Brown, Esq., 10s. 6d. ; Dr. Mackintosh, M.V.O., 10s. ; Miss Marwick, 10s. ; Miss Donald, 3s. 6d.). Cardiff Infirmary (per Mr. L. D. Rea), £13 10s. (viz. "Well Wisher," £2 ; Lady Aberdare, £1 1s. ; Major-General H. H. Lee, £1 1s. ; Lieut.-Colonel Bruce Vaughan, £1 1s. ; Mrs. Nixon, £1 1s. ; Mrs. Brockett Grover, £1 1s. ; Mr. Wm. Jas. Thomas, £1 1s. ; Mr. E. H. Ebsworth, £1 1s. ; Mr. H. Woolcott Thompson, £1 1s. ; Mr. Isaac Samuel, £1 1s. ; smaller amounts, £2 1s.) ; Stewart Garnett, Esq., £2 2s. ; Nurse Gutteridge, 3s. ; Nurse F. J. Roberts, 2s. 6d. ; Nurse Eveson, 1s.

Received by Mr. G. Q. Roberts, Hon. Secretary : Miss Tidswell, £20 ; W. F. Courthope, Esq., £10 ; S. A. P. Kitcat, Esq., £5 5s. ; Mrs. John Bell, £5 ; Arthur Young, Esq., £5 ; Lady Camilla Fortescue, £5 ; "In memory of Sir Chas. Daubeney, G.C.B., of the 55th Foot," £5 ; Lord and Lady Archibald Campbell, £5 ; Miss Myrtis Halford's collection, £3 10s. ; Mrs. Thomas-Salford, £3 3s. ; Hon. L. W. Rothschild, £2 2s. ; W. G. Raphael, Esq., £2 2s. ; R. G. Hargreaves, Esq., £2 ; Lady Helen Munro Ferguson, £2 ; Sir Michael Culme-Seymour, G.C.B., G.C.V.O., £2 ; "Florence," £1 1s. ; Mrs. Sclater, £1 1s. ; Hon. John Ferguson, C.M.G., £1 1s. ; Mrs. Barry Hart, £1 1s. ; Major-General H. S. Gough, £1 1s. ; Rowland Ward, Esq., £1 1s. ; H. C. Pilkington, Esq., £1 1s. ; Miss Ramsay L'Amy, £1 1s. ; Miss Baily, £1 1s. ; Miss V. Chapman, £1 ; Mrs. Goddard, £1 ; Mrs. Algernon Thynne, £1 ; Mrs. Thorburn, £1 ; Rear-Admiral King Hall, £1 ; Colonel P. E. Hill, £1.

Contributions may be sent to the Hon. Secretary, Florence Nightingale Memorial, St. Thomas's Hospital, Albert Embankment, London, S.E., or to the Editor of The Hospital, 29 Southampton Street, Strand, London, W.C.

### The Week's Novelties.

### THE WHISKEY FOR HOSPITAL MEN.

Daniell Crawford, 81 Queen Street, Glasgow, the noted manufacturer of fine Scotch whiskey, has sent us a sample of his products. The whiskey is genuine Scotch, of pleasant flavour, and possesses those relative food qualities which alcohol in certain cases may be said to give in such standard drinks, for instance, as beer and whiskey. Whiskey is a beverage the value of which depends largely on the purity of its ingredients, and, without enlarging on the analytical nature of Crawford's, it is safe to recommend it as a good type of matured spirit. In this sense it is worth the careful attention of all institutional workers, whether for certain hospital purposes or as a beverage which may be included very usefully in the Christmas hamper, which is gaining in popularity as a present at this season.

